# Surface Properties of Recombinant Pea Vicilin and Cupin-1.2 Solutions in 8M Urea

**DOI:** 10.3390/polym17182463

**Published:** 2025-09-11

**Authors:** Nikolay Isakov, Dmitry Angel, Mikhail Belousov, Giuseppe Loglio, Reinhard Miller, Anton Nizhnikov, Boris Noskov

**Affiliations:** 1 St Petersburg State University, 199034 St. Petersburg, Russia; st055657@student.spbu.ru (N.I.); st117542@student.spbu.ru (D.A.); belousovmix@gmail.com (M.B.);; 2 All-Russia Research Institute for Agricultural Microbiology, 196608 St. Petersburg, Russia; 3 Institute of Condensed Matter Chemistry and Technologies for Energy, 16149 Genoa, Italy; giuseppe.loglio@ge.icmate.cnr.it; 4 Institute of Condensed Matter Physics, Technical University Darmstadt, 64289 Darmstadt, Germany; reinhard.miller@pkm.tu-darmstadt.de

**Keywords:** vicilin, cupin-1, protein aggregates, urea, dilational dynamic surface elasticity, surface pressure, adsorption kinetics

## Abstract

The kinetic dependencies of the surface pressure, the dilational dynamic surface elasticity and ellipsometric angles of cupin-1.2 and vicilin solutions in 8M urea were measured at different concentrations. The analysis of these kinetics dependencies and the obtained master curves allowed us to determine a few adsorption steps in the investigated systems and showed that the master curves are individual characteristics of the protein for a given solvent. At the same time, the shape of these curves can be different for adsorbed and spread layers of plant proteins indicating different structures of these layers. The dependencies of the dynamic surface elasticity on surface pressure are non-monotonic, unlike the corresponding results for most of the solutions of the investigated plant proteins. The extremums of these dependencies can be connected to the formation of the distal region of the surface layer in agreement with the theory for the surface viscoelasticity of polymer solutions.

## 1. Introduction

Plant proteins have attracted significant attention recently because of the attempts to substitute proteins of animal origin, which are more expensive and harmful for the environment [1,2,3,4,5,6,7,8]. Although plant proteins and their aggregates similar to their animal counterparts can be effective stabilizers of foams and emulsions, can contribute to gelation, enhancing quality of various products [6,7,8,9,10], or find applications in drug delivery systems [11,12], the broad investigation of the properties of their solutions and aggregate dispersions has started only recently [1,2,3,6,7,8,13,14,15,16,17,18,19,20,21,22,23,24,25]. Possible reasons for the limited applications of plant proteins in the past consist of their low solubility in water in a broad pH range and somewhat worse functional properties than those of animal proteins.

The surface activity of the proteins and the surface properties of their solutions are of special importance for numerous applications, for example, for the stabilization of foams and emulsions [26]. For this reason, recent studies have focused on solutions of plant seed storage proteins and dispersions of plant protein aggregates.

A great part of these studies concentrated on legume proteins [1,19,22,23,27] and wheat proteins [13,16,21]. These proteins are important because they can be obtained from industrial by-products.

Poirier et al. have applied a wide range of the methods of surface chemistry to the investigation of plant protein adsorption and the surface properties of their solutions [16,17,18]. Meanwhile Sagis et al. have intensively studied the surface rheological properties of solutions of plant proteins [5,23,27,28]. Though both groups used the methods of surface dilational rheology, they focus on different aspects of the plant protein behavior at liquid–fluid interfaces.

Most publications in this field deal with protein mixtures, in particular, with protein isolates, but not with highly purified individual proteins. The analysis of the surface properties of protein solutions is more difficult in the former case of more complex systems, indicating the importance of protein purification and characterization.

As an alternative to a conventional method of protein extraction from plants, the microbiological techniques can produce proteins of higher purity. Using these recombinant proteins helps to determine correlations between the protein structure, the solution composition, its properties, and the aggregation behavior.

Antonets et al. have recently investigated the amyloid fibril formation of the garden pea (*Pisum sativum* L.) storage globulin vicilin, highlighting the role of the conserved β-barrel domains of the cupin-1 family, namely cupin-1.1 and cupin-1.2, in fibril assembly [14,29]. Recent findings demonstrate that cupin-1.1 forms aggregates in the surface layer, even in the presence of 8M urea [24,30].

The aim of this work is to investigate the adsorption layer formation of cupin-1.2 and vicilin solutions in 8M urea and to estimate the changes in the layer structure at an increase in the protein concentration. The comparison of the adsorption layer properties of these two proteins with those of the layers of previously studied cupin-1.1 allows further elucidation of the layer properties of plant proteins. Special attention is paid to the surface dilational rheological properties, especially the dilational surface elasticity. This parameter determines, in particular, the evolution of thin foam films, and thereby controls the growth and destruction of air bubbles in foams [26,31]. The determination of the surface elasticity can help to predict the stability of liquid phase disperse systems.

## 2. Materials and Methods

### 2.1. Materials

Recombinant cupin-1.2 and vicilin C-terminally fused with a 6x-His tag were expressed using previously constructed plasmid and *E. coli* strain BL21 (DE3) (New England Biolabs, Ipswich, MA, USA) as described elsewhere [14]. The proteins were purified using affinity chromatography for further work. Cupin-1.2 and vicilin were dissolved in 8M aqueous urea with TrisHCl buffer (20 mM, pH 8.0) and stored in a refrigerator. The stock solution was diluted before measurements to the required concentration by 8 M urea solution in water. Note that urea changes the solution ionic strength to a lesser extent than guanidine hydrochloride.

Urea (LenReactiv, St Petersburg, Russia) was recrystallized from its solution in ethanol and after that from its 70 *v*/*v* % ethanol aqueous solution. Ethanol (LenReactiv, Russia) was distilled one time and deionized water was distilled two times in a glass apparatus before use.

### 2.2. Methods

Surface tension was measured by the Wilhelmy plate technique using a platinum plate with a roughened surface to improve wetting. The accuracy of the measurements was approximately ±0.3 mN/m.

The oscillating barrier technique was used to determine the dilational dynamic surface elasticity using an ISR instrument (KSV NIMA, Finland) described in detail elsewhere [32,33]. Two Teflon barriers moved back and forth along polished brims of a Teflon Langmuir trough with a frequency of 0.03 Hz. The amplitude of the surface area oscillations was 2%. The platinum plate was placed in the center of the Langmuir trough and aligned orthogonally to the main axis of the trough. The complex dilational surface elasticity E was calculated as the ratio of the complex amplitudes of the surface tension δγ and the relative surface area δ ln A:

Ε=Re(Ε)+i Im(Ε)=δγ/δ ln A

The elasticity modulus equals the ratio of the oscillation amplitudes, while the phase shift between the oscillations of the two parameters provides the phase angle and thereby allows determination of the real $Re\left(Ε\right)$ and imaginary $Im\left(Ε\right)$ components of the dynamic surface elasticity. The accuracy of the surface elasticity determination was close to ±5%. Only the real part of the surface elasticity was discussed in detail below because its imaginary part proved to be insignificant in comparison to the real part.

A null ellipsometer (Optrel-GBR, Germany) with a laser wavelength of 623.8 nm was used to analyze the kinetics of protein adsorption. The measurements were taken at an angle of incidence near 50°, i.e., close to the Brewster angle. The difference in the ellipsometric angle ∆ of the solutions under investigation and the value for the pure solvent ∆_0_ as presented below are expected to be proportional to the surface concentration of the adsorbed protein [34].

The morphology of the spread layers of cupin-1.2 and vicilin was determined by atomic force microscopy (AFM) using the NTEGRA Prima instrument (NT-MDT, Zelenograd, Russia). The spread layer was transferred from the aqueous surface onto a freshly cleaved mica plate using the Langmuir–Schaeffer method and dried in a desiccator. The AFM imaging was conducted in a semicontact regime, using a cantilever with an approximate curvature radius of 10 nm.

**Figure 1:**
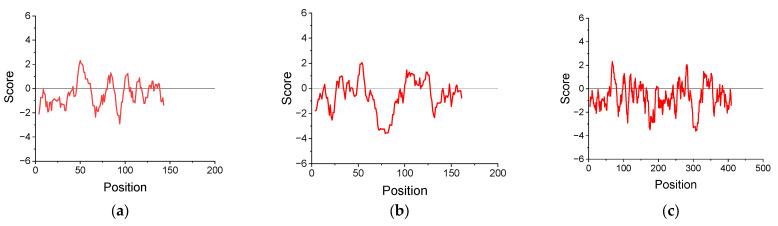
Hydropathy plot for cupin-1.1 (**a**), cupin-1.2 (**b**), and vicilin (**c**) calculated by the Kyte and Doolittle method.

The change in hydropathy along the protein chain was assessed using the Kyte and Doolittle algorithm assigning a hydropathy index to each amino acid residue (negative values indicate hydrophilicity; positive values indicate hydrophobicity) [35]. A sliding window of nine residues was applied by averaging the hydropathy score for each residue and its eight adjacent neighbors. The hydropathy plots for cupin-1.1, cupin-1.2, and vicilin were calculated using the amino acid sequences given in Ref. [14] (Figure 1).

The grand average of hydropathy (GRAVY) was calculated by averaging the hydropathy indices across the entire protein sequence (Table 1). Additionally, the aliphatic index (AI), which reflects the relative volume occupied by aliphatic acids (alanine, valine, leucine, and isoleucine), was calculated as a weighted sum of the mole fractions of these residues taking into account their relative volumes as described in Ref. [36] (Table 1).

## 3. Results

### 3.1. Cupin-1.2 Adsorption Kinetics

**Figure 2:**
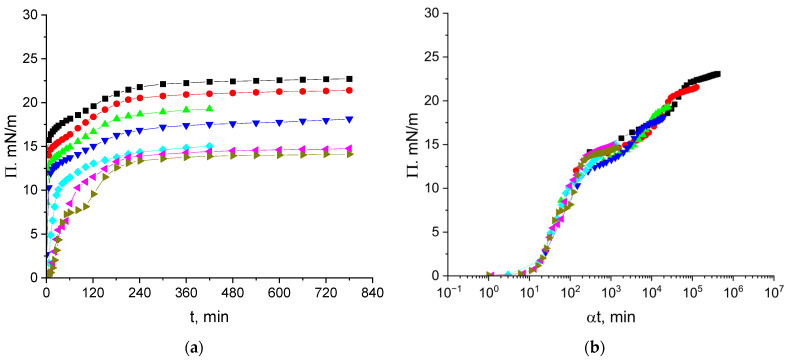
Kinetic dependences of the surface pressure (**a**) and master curve of these dependencies (**b**) for cupin-1.2 solutions in 8M urea at concentrations of 1 (olive triangles), 2.5 (magenta triangles), 5 (cyan diamonds), 10 (blue triangles), 20 (green triangles), 50 (red circles), and 100 mg/L (black squares). Reference concentration is 1 mg/L.

The kinetic dependencies of the surface pressure of cupin-1.2 solutions in 8M urea (pH 8) are shown in Figure 2a. The steady state value is reached after approximately 5 h for all the concentrations and is close to 14 mN/m at concentrations from 1 to 5 mg/L but increases at higher concentrations up to 22 mN/m at a concentration of 100 mg/L. The concentration dependences of the surface pressure of cupin-1.2 and vicilin solutions in 8M urea are qualitatively similar to the corresponding results for cupin-1.1 solutions in the investigated concentration range (Supplementary Materials Figure S1) [30].

The kinetic dependencies in Figure 2a collapse into a single master curve (Figure 2b), with a shift factor α depending on the concentration (Figure S2a). The master curve analysis has been applied only recently to the adsorption kinetics of proteins [16,17,25,37]. This approach consists of the shifting of all the kinetic curves to a single reference curve, thereby excluding the concentration as an additional variable. The time–concentration superposition assumes a mathematical equivalence between the time and concentration effects, allowing a kinetic curve for a reference concentration $c_{ref}$ to be reconstructed from other concentrations $c_{i}$ using the shift factors $\alpha \left(c_{i}\right)$:

(1) $$\Pi \left(c_{i},t\right)=\Pi \left(c_{ref},t_{ref}\right),$$

where $t_{ref}=\alpha \left(c_{i}\right)t$.

This procedure generates a unified curve–master curve, which effectively extends the observable adsorption window and highlights kinetic steps as the changes in its shape. Poirier et al. showed that the dependence of the shift factor on concentration differed for the diffusion and barrier-controlled adsorption mechanisms [16]. In the latter case, it is a linear dependence:

(2) $$\alpha \left(c_{i}\right)=\frac{c_{i}}{c_{ref}},$$

while it is a quadratic dependence in the former case:

(3) $$\alpha \left(c_{i}\right)={\left(\frac{c_{i}}{c_{ref}}\right)}^{2},$$

**Figure 3:**
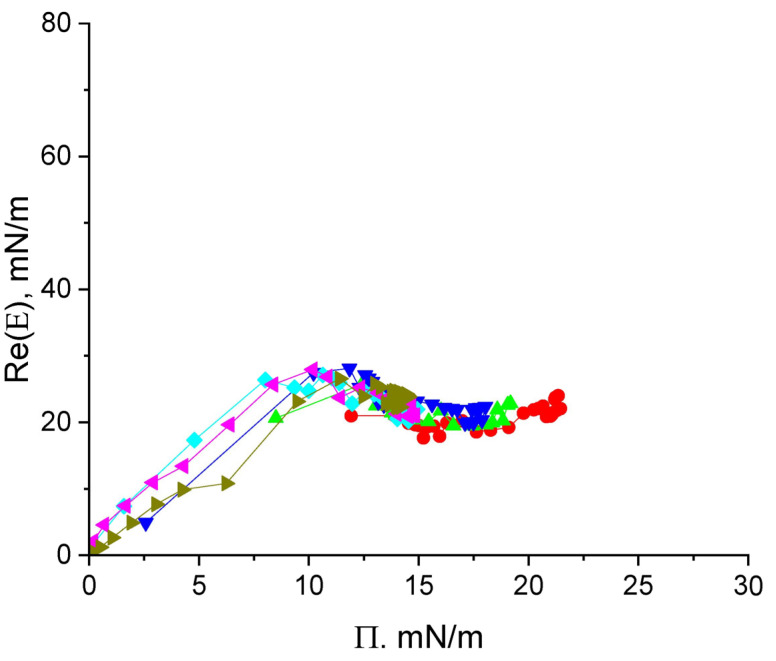
The real part of the surface elasticity of cupin-1.2 solutions in 8M urea at concentrations of 1 (olive triangles), 2.5 (magenta triangles), 5 (cyan diamonds), 10 (blue triangles), 20 (green triangles), and 50 mg/L (red circles) as a function of surface pressure.

Figure 2b displays a short induction period (~15 min) corresponding to negligible interactions between the protein molecules or their aggregates in the surface layer at the beginning of adsorption. The subsequent increase in the interactions results in a gradual increase in the surface pressure up to approximately 13 mN/m. After that, the surface pressure starts to increase more slightly up to about 16 mN/m. This quasi-plateau regime can correspond to the formation of a distal region of the surface layer (cf. Figure 3). After the quasi-plateau, the surface pressure increases stronger again up to about 24 mN/m, indicating that an increase in the concentration of hydrophobic groups in the proximal region of the surface layer leads to stronger changes in the layer structure (Figure 2b).

The dependence of the shift factor α on concentration is non-linear but the exponent is less than 2, presumably indicating a mixed adsorption mechanism (Figure S2a).

### 3.2. Dynamic Surface Elasticity of Cupin-1.2 Solutions

The dynamic surface elasticity of cupin-1.2 solutions in 8M urea changes non-monotonically with the surface age and surface pressure (Figure 3 and Figure S3A). Similar non-monotonic dependencies have been observed earlier for solutions of nonionic amphiphilic polymers and unfolded protein molecules [32,38,39,40].

The whole graph can be divided into three distinct regions. At surface pressures less than approximately 8 mN/m, the surface elasticity increases approximately proportionally to the surface pressure with a slope of ~3 and reaches ~27 mN/m in a local maximum. This behavior indicates an increase in the segment interactions within an almost two-dimensional layer [41].

At higher surface pressures, the dynamic surface elasticity goes through a local maximum and starts to decrease slightly after that (Figure 3). This behavior indicates the formation of a distal region of the surface layer (the region of loops and tails) and a new mechanism of the surface stress relaxation: the segment exchange between the distal and proximal regions of the surface layer in the course of the layer compression and expansion (cf. section Discussion) [32,40]. The relatively low surface elasticity (~20 mN/m) at surface pressures 14–16 mN/m beyond the local maximum corresponds to the quasi-plateau region in Figure 2b.

At even higher surface pressures (>17 mN/m), the surface elasticity increases almost linearly with the surface pressure. The layer thickening can occur at this step as it was described for solutions of β-casein by Douillard et al. [41]. The relation between the real and imaginary parts of surface elasticity appears to be almost constant at all surface pressure values obtained (Figure S4).

### 3.3. The Kinetic Dependencies of the Ellipsometric Angle Δ of Cupin-1.2 Solutions

**Figure 4:**
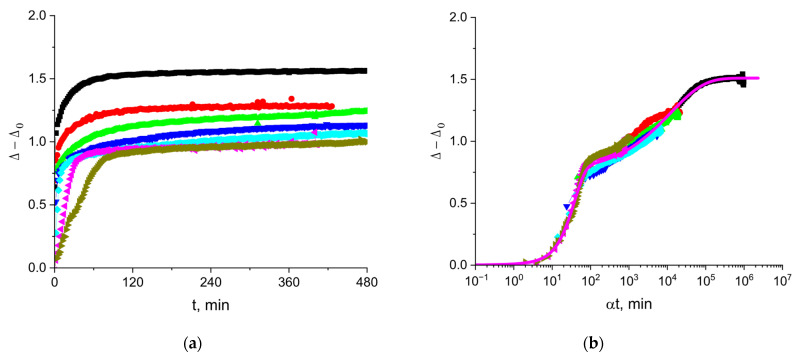
Kinetic dependences of angle $∆-{∆}_{0}$ (**a**) and the master curve of these dependencies (**b**) for solutions of cupin-1.2 in 8M urea at concentrations of 1 (olive triangles), 2.5 (magenta triangles), 5 (cyan diamonds), 10 (blue triangles), 20 (green triangles), 50 (red circles), and 100 mg/L (black squares).

The ellipsometric angle Δ is approximately proportional to the surface concentration of an amphiphilic substance [34]. As a result, Δ for cupin-1.2 solutions starts to deviate from the value for water at shorter surface lifetimes than the surface tension and starts to increase even when the surface pressure remains negligible (Figure 4). The steady-state values of the angle Δ increase gradually at the increase in the protein concentration from 1 to 50 mg/L, and rather abruptly at higher concentrations from 50 to 100 mg/L. The use of the shift factor α allows construction of a master curve, as shown in Figure 4b. The gradual change in the steady-state values can be connected to the formation of a quasi-plateau region on the master curve, while the sharp increase corresponds to the subsequent increase in Δ. The layer thickening can lead to an increase in the angle beyond the quasi-plateau state. Note that the kinetic master curve of Δ follows the same trend as the kinetic master curve for surface pressure (Figure 2b). These results imply that the increase in the surface concentration in the course of adsorption is the main factor determining the changes of the surface properties, even at high surface pressures.

The magenta line in Figure 4b presents the results of calculations according to the following equation:

(4) $$∆-{∆}_{0}=0.75*\left(1-e^{-0.0042*{\left(\alpha t\right)}^{1.5}}\right)+0.76*\left(1-e^{-0.009*{\left(\alpha t\right)}^{0.5}}\right),$$

The reference concentration for the master curve is 1 mg/L. This approximation indicates two main steps of the kinetic dependencies, which can presumably correspond to the formation of a quasi-monolayer and a thicker layer after that.

### 3.4. Vicilin Adsorption Kinetics

**Figure 5:**
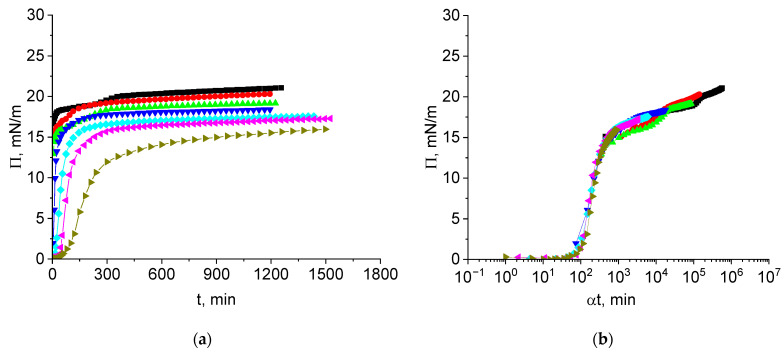
Kinetic dependences of the surface pressure (**a**) and master curve of these dependencies (**b**) for vicilin solutions in 8M urea at concentrations of 1 (olive triangles), 2.5 (magenta triangles), 5 (cyan diamonds), 10 (blue triangles), 20 (green triangles), 50 (red circles), and 100 mg/L (black squares). Reference concentration is 1 mg/L.

The kinetic dependencies of the surface pressure of vicilin solutions in 8M urea are slightly different from those for cupin-1.2 solutions (Figure 5a). The steady state values of the surface pressure do not differ by more than 5 mN/m in a broad concentration range from 2.5 to 100 mg/L and increase gradually with the concentration.

**Figure 6:**
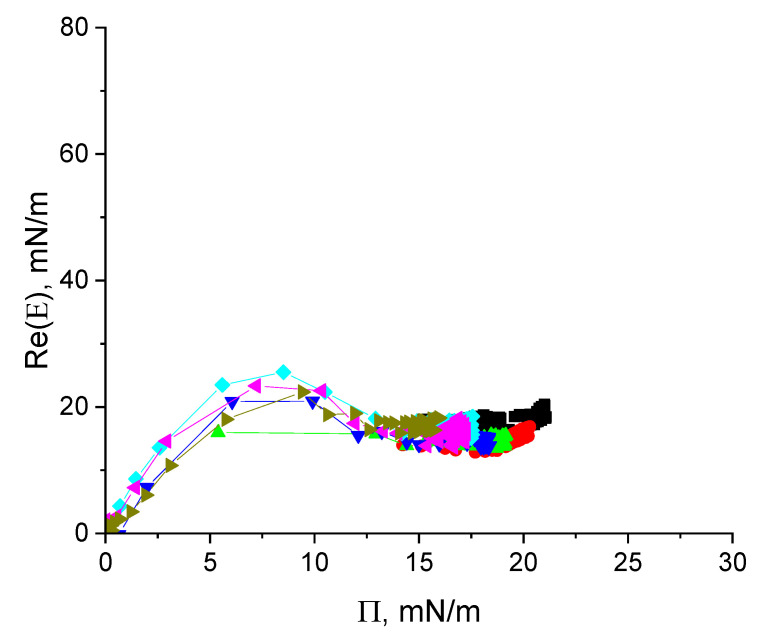
The real part of surface elasticity of vicilin solutions in 8M urea at concentrations of 1 (olive triangles), 2.5 (magenta triangles), 5 (cyan diamonds), 10 (blue triangles), 20 (green triangles), 50 (red circles), and 100 mg/L (black squares) as a function of surface pressure.

All the kinetic dependencies collapse into a single master curve if a shift factor α is introduced to obtain the normalized time (Figure 5b). This master curve reveals again three distinct regions similar to the results for cupin-1.2 solutions (Figure 2b). The first region is an induction period of about 80 min. The second region is characterized by significant growth of the surface pressure up to approximately 15 mN/m. In contrast to the results for cupin-1.2 and similarly to those for cupin-1.1, this region corresponds to a significant increase in the surface elasticity and its subsequent decrease (cf. Figure 6) [30]. Note that the elasticity growth is sharper for vicilin than for cupin-1.2 adsorption layers. The third region of the master curve corresponds to a slighter and almost linear increase in the surface pressure.

The shift factor exhibits an approximately linear dependence on concentration in agreement with the barrier-controlled adsorption mechanism (Figure S2b) similar to the data for cupin-1.1 solutions [30].

### 3.5. Dynamic Surface Elasticity of Vicilin Solutions

The surface elasticity of vicilin solutions is an almost linear function of the surface pressure with a slope of ~4 for Π < ~6 mN/m (Figure 6). At higher surface pressures, the surface elasticity reaches a local maximum of about 24 mN/m. The surface elasticity decreases after that presumably due to the formation of a distal region of the surface layer.

The small difference between the surface elasticities in the regions of the local maximum and the subsequent local minimum (~8 mN/m) (Figure 6 and Figure S3B) presumably indicates that vicilin preserves some elements of its tertiary structure even in the presence of a strong denaturant. At surface pressures from 15 to 22 mN/m, the surface elasticity increases slightly with a slope close to 1 for all investigated protein concentrations (Figure 6).

The ratio between the imaginary and real components of surface elasticity remains small and close to the values for cupin-1.2 solutions (Figure S4b).

### 3.6. The Kinetic Dependencies of the Ellipsometric Angle Δ of Vicilin Solutions

**Figure 7:**
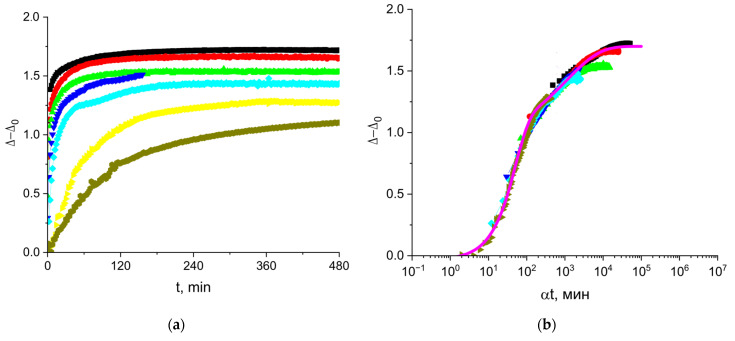
Kinetic dependences of the ellipsometric angle $∆-{∆}_{0}$ (**a**) and the master curve (**b**) for solutions of vicilin in 8M urea at concentrations of 0.5 (olive hexagons), 1 (yellow triangles), 5 (cyan diamonds), 10 (blue triangles), 20 (green triangles), 50 (red circles) and 100 mg/L (black squares). Reference concentration for master curve is 1 mg/L.

The ellipsometric angle Δ changes with the surface age at the very beginning of the vicilin adsorption when the surface pressure remains negligible, and the kinetic dependencies of Δ (Figure 7) do not display an induction period unlike the kinetic dependencies of the surface pressure (Figure 5b). The ellipsometric angle Δ reaches a steady state value for 120 min or less at the concentrations above 5 mg/L (Figure 7a). This time is noticeably shorter than that for the kinetic dependencies of the surface pressure. Despite the observed differences, the kinetic dependencies of the surface tension and the ellipsometric angle exhibit similar trends. The latter dependencies at different concentrations also lead to a master curve if the angle Δ is plotted as a function of the normalized time (Figure 7b).

The fitting of the master curve by a sum of two exponents (magenta line in Figure 7b) indicates a two-step adsorption process similar to solutions of cupin-1.2:

(5) $$∆-{∆}_{0}=1.00*\left(1-e^{-0.015*{\left(\alpha t\right)}^{1.2}}\right)+0.75*\left(1-e^{-0.03*{\left(\alpha t\right)}^{0.5}}\right)$$

### 3.7. Dynamic Properties and Morphology of Cupin-1.2 and Vicilin Spread Layers

**Figure 8:**
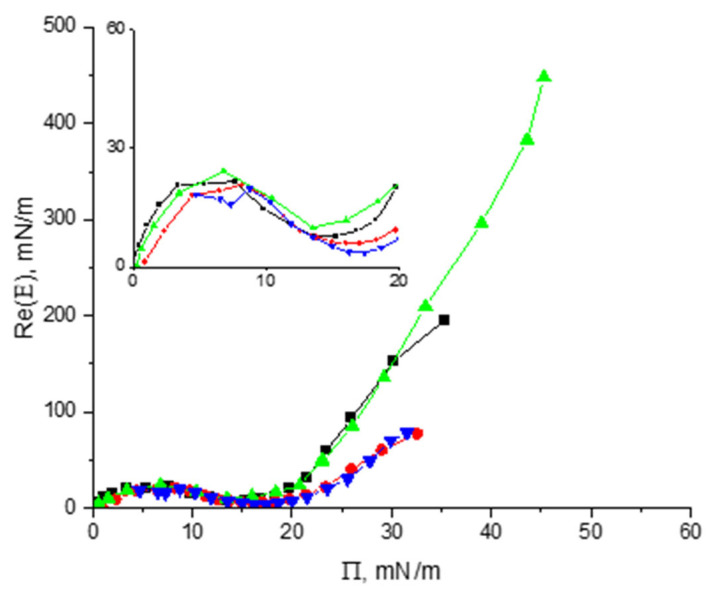
The real part of the surface elasticity of spread layers of cupin-1.2 and vicilin on the surface of water (green triangles and black squares, respectively) and 8M urea (blue triangles and red circles, respectively).

Vicilin and cupin-1.2 were spread onto the surface of both pure water and 8M urea from their solutions in 8M urea. After that, the spread layers were compressed and the dynamic surface elasticity was measured as a function of the surface pressure leading to similar results for both proteins (Figure 8).

The surface elasticity increases at the beginning, reaches approximately 22 mN/m at a surface pressure of ~6 mN/m, and decreases after that to ~8 mN/m on the water surface and to ~5 mN/m on the surface of the urea solution. The local minima of the surface elasticity correspond to surface pressures of 14 mN/m and 17 mN/m, respectively. Beyond the region of the local minimum, the surface elasticity starts to increase again with a steeper slope for the layers on water surface than on the surface of 8M urea solution.

The significant overlap of the curves in Figure 8 for vicilin and cupin-1.2 layers at surface pressures less than 20 mN/m indicates similar intermolecular interactions in the spread layers on different substrates and their similar structural organization. Cupin-1.2 domains presumably contribute to the formation of a relatively soft corona of the vicilin aggregates leading to the same initial peaks of surface elasticity for the layers of both proteins.

Atomic force microscopy shows that at low surface pressures (Figure S5a,e), both cupin-1.2 and vicilin layers on a water surface are strongly heterogeneous with the averaged thickness close to 1 nm. The further compression results in the formation of a network of thin aggregates at the interface with relatively rare and thicker particles in it (Figure S5b,c,f,g).

## 4. Discussion

The application of the normalized time αt gives a possibility to construct a single master curve from different kinetic dependencies Π (t) for various concentrations of a given protein (Figure 2b and Figure 5b). In spite of a certain similarity, the shapes of the master curves for cupin-1.2 and vicilin are not the same. They also differ from the corresponding results for solutions of another recombinant protein cupin-1.1 studied earlier [30].

Recently, similar master curves have also been obtained for solutions of a few isolates of the following plant proteins: gliadin [16], sunflower protein [17], pea, soy, rice, mung bean proteins [25], and also of the complexes of lupin protein and pectin [37]. The results for recombinant proteins in this study are not significantly different from those for protein isolates and can be considered together with them.

The shape of the master curve is determined mainly by the protein. For example, the master curve for pea protein isolate [25], which contains mainly vicilin, is similar to that for the recombinant vicilin in this study (Figure 5b) despite the fact that the former protein contains components of different molecular weight.

Although transition from the kinetic dependencies of the surface pressure on the surface age to the dependencies on the normalized time seems to be simple, it has not been substantiated strictly yet on the basis of the kinetic equations of adsorption, except in the case of the diffusion-controlled adsorption at small surface pressures [16]. Therefore, this approach can be considered as an empirical one.

It is noteworthy that despite the fact that the master curves for different plant protein solutions have some common features, they differ from one another and thereby are individual characteristics of the protein for a given solvent. The shapes of the master curves are approximately the same only at low surface pressures when a rather fast increase in the surface pressure occurs after a short induction period (usually 5–100 min) corresponding to almost zero surface pressures [16,17,25,30,37].

The duration of the induction period can depend on the diffusion rate to the interface and thereby on the molecular weight. It is higher (~80 min) for the heavier vicilin than for the lighter cupin-1.2 (15 min) and cupin-1.1 (40 min).

At low surface pressures, the shift factor is usually a quadratic function of the concentration in agreement with the diffusion-controlled adsorption mechanism [16,25]. Another argument in favor of this mechanism is the square root dependence of the surface pressure on the surface age (Figure S6). This feature is in agreement with the Ward–Tordai equation for small surface concentrations [42].

It is also possible to obtain master curves by plotting the ellipsometric angle as a function of the normalized time (Figure 4b and Figure 7b) with a shape similar to that of the corresponding curves for the surface pressure (Figure 2b and Figure 5b). This similarity is obviously a consequence of an almost linear relation between the angle Δ and the surface concentration [34].

The ellipsometric master curves in Figure 4b and Figure 7b can be approximated by a sum of a stretched exponent and compressed one (Equations (4) and (5)). The two terms in these equations do not relate to two different adsorption mechanisms. The first modified exponent covers, in particular, the range of normalized times corresponding to the violation of the diffusion-controlled adsorption mechanism. Presumably, the first compressed exponent can be related mainly to the increase in the surface concentration in the course of the proper adsorption process. The second stretched exponent can be related mainly to the subsequent slow changes in the surface layer structure and conformational transitions of the adsorbed protein molecules.

The rate of the surface pressure change observed for vicilin solutions in this work significantly exceeds that observed by Chang et al. for the solutions of pea vicilin isolate [2]. This difference is presumably a consequence of the high urea concentration in the given study. The partial protein unfolding under the influence of urea can increase the protein surface activity and therefore the adsorption rate [32].

Another master curve appears if the real part of the dilational dynamic surface elasticity or the surface elasticity modulus are plotted as a function of the surface pressure (Figure 3, Figure 6 and Figure 8). In this case, the experimental data for different concentrations fall on a single curve. This master curve is also an individual characteristic of a substance for a given solvent. Its shape reflects the organization of the adsorption layer and thereby the properties of macromolecules.

The master curves were constructed first for solutions of amphiphilic polymers [40,43,44] and proteins [32,39,41]. For dispersions of hard and soft nanoparticles, for example for globular protein solutions, the dependencies of the surface elasticity on surface pressure are usually monotonic but have one or two local maxima if the systems contains flexible chains [32,40].

The flexibility of adsorbed protein molecules, at least their parts, can be a consequence of their intrinsic disorder, an impact of strong denaturants as in the systems under investigation or can arise in the course of adsorption. The third possibility is frequently discussed in the literature [45]. The non-monotonic kinetic dependencies of the dynamic surface elasticity arise if the flexible chains of macromolecules start to form loops and tails in the distal region of the surface layer. In this case, the surface stresses can be relaxed at the expense of the segment exchange between the proximal and distal regions of the surface layer in agreement with the theory of the dilational surface viscoelasticity of polymer solutions [40,46], and the dynamic surface elasticity decreases after a local maximum.

It is also possible to observe local maxima of the surface elasticity when some aggregates are formed in the surface layer and the surface stresses can be relaxed at the expense of the mass exchange between these aggregates and the surrounding two-dimensional phase or when there is a transition between a monolayer and a bilayer, or a multilayer, and the stress relaxation occurs as a result of the segment exchange between different layers [47].

Note that the non-monotonic dependencies of the dilational surface elasticity on surface pressure were also observed for dispersions of polymer microgels if a relatively hard core of microparticles is surrounded by a soft corona, which is formed by a flexible chain [48,49,50]. In this case, the dependencies of the surface elasticity on surface pressure for microgel dispersions can be very close to those for solutions of amphiphilic macromolecules [48].

The results for adsorption and spread layers of the most of plant proteins show that the dilational surface elasticity at the liquid–air interface usually increases monotonically with the surface pressure, as in the case of globular solutions of animal proteins, thereby indicating that the macromolecules are not unfolded in the surface layer [2,51,52,53,54,55,56]. This conclusion is in agreement with the low solubility of plant proteins.

The adsorbed and spread layers of cupin-1.1 [24,30] and of the proteins studied in this work are characterized by a different behavior, and the dynamic surface elasticity is a non-monotonic function of the surface pressure. At low surface pressures, <9 mN/m for cupin-1.2 solutions and Π <6 mN/m for vicilin solutions, the dilational dynamic surface elasticity is almost proportional to this parameter. The protein molecules are spread along the surface and presumably form a monolayer as in the case of β-casein adsorption layers [41].

The subsequent decrease in the surface elasticity after a local maximum can be connected to the formation of loops-and-tails in the distal region of the surface layer by some flexible parts of the protein molecules. The AFM images do not indicate the formation of unstable surface aggregates in the region of the maximum of the dynamic surface elasticity (Figure S5) and another interpretation of the surface elasticity maxima is less probable. The relatively free protein chains in the surface layer can be either partially unfolded protein molecules or some parts of soft coronas of the existing protein aggregates as in the case of cupin-1.1 solutions [30].

The elasticity maximum occurs at lower surface pressures for solutions of the more hydrophilic vicilin molecules (~8 mN/m) than for solutions of cupin-1.1 and cupin-1.2, in agreement with the lower value of the GRAVY index for vicilin (Table 1). It is possible to assume that the layer of more hydrophobic macromolecules can sustain greater surface pressures before the formation of loops-and-tails as compared with a relatively more hydrophilic vicilin layer (Figure 1).

The dynamic surface elasticity of vicilin and cupin-1.2 solutions decreases not strongly after the elasticity maximum, by 40% and 30%, respectively. This decrease is similar to that for the solutions of animal globular proteins but much less than for the solutions of amphiphilic polymers [43,44]. The surface elasticity in the region of the local minimum is connected presumably to the flexibility, size and the local concentration of relatively free chains of macromolecules. It is low (<10 mN/m) for cupin-1.1 solutions in 8M of a strong denaturant [30]. Urea unfolds the protein globules and, at the same time, destroys the protein aggregates.

This effect is not very strong for cupin-1.2 and vicilin, but much stronger for more hydrophobic cupin-1.1 (Table 1). Cupin-1.1 can form larger aggregates in solutions in pure water and the local elasticity minimum in this solvent exceeds 30 mN/m [30]. The denaturant in the subphase also influences the properties of spread protein layers. If the subphase contains urea, the dynamic surface elasticity in the region of the local minimum becomes lower (Figure 8).

The difference between the properties of spread layers on the surface of water and 8M urea increases significantly at surface pressures beyond the region of the elasticity minimum when the surface elasticity in the former case reaches ~ 450 mN/m, while in the latter case, it is much lower at <80 mN/m (Figure 8). This distinction can be caused by the destruction of large aggregates and the formation of more extended coronas under the influence of urea.

The surface properties beyond the elasticity minimum are determined mainly by the interactions between hard cores of the aggregates. Simultaneously, the thickness of the adsorption layer can increase and it cannot be considered as a monolayer. The protrusion of hydrophobic protein groups into the air phase at high urea concentrations can also lead to the increase in the repulsion between macromolecules at high surface pressures [57], and thereby to an increase in the dynamic surface elasticity.

## 5. Conclusions

Unlike the proteins extracted from plants, their counterparts are characterized by high purity and exact values of the molecular weight. Therefore, this study uses this relatively simple model system to examine recently discovered peculiarities of the adsorption of plant proteins at the solution–air interface and to elucidate the adsorption mechanism. The detailed investigation of the adsorption kinetics of recombinant vicilin and cupin-1.2 allowed us to discover a few distinct steps. This process is controlled by the diffusion from the bulk to the interface only at the very beginning at low surface pressures. Nevertheless, the use of the normalized time allowed us to construct a single kinetic master curve for the whole range of the investigated adsorption times. This peculiarity of the kinetic dependencies has also been discovered recently for the solutions of cupin-1.1 and some other plant proteins. Moreover, similar master curves were also obtained for the ellipsometric angle Δ for the first time according to our knowledge. The master-curves are different for different plant proteins and their shape depends on the applied solvent. If the solvent is the same, they can be considered as individual characteristics of the protein. Their shape is determined by the packing of macromolecules in the surface layer and therefore of the protein structure. The plotting of the dilational dynamic surface elasticity as a function of the surface pressure leads to another master curve. Its shape is also characteristic of a given protein for the same solvent but also depends on the way of preparation of the protein layer: the curves for adsorbed and spread layers can differ noticeably. The dependencies of the dynamic surface elasticity on surface pressure for the layers of vicilin, cupin-1.2 and recently studied cupin-1.1 have a local maximum and a local minimum and thereby differ significantly from the monotonic dependencies for other studied plant proteins. The interpretation of the extremums of the obtained master curves is possible on the basis of the formation of the distal region of the surface layer in agreement with the theory of the surface viscoelasticity of polymer solutions [46].

## Figures and Tables

**Table 1:** Characteristics of cupin-1.1, cupin-1.2 and vicilin.

Characteristic	Cupin-1.1	Cupin-1.2	Vicilin
Aliphatic index	96.22	83.31	84.28
GRAVY	−0.495	−0.640	−0.744

## Data Availability

The original contributions presented in this study are included in the article/Supplementary Materials. Further inquiries can be directed to the corresponding author.

## Supplementary Materials

The following supporting information can be downloaded at https://www.mdpi.com/article/10.3390/polym17182463/s1: Figure S1: Surface pressure at surface age of 420 min as a function of the concentration of cupin-1.1 (black squares), cupin-1.2 (red circles), and vicilin (green triangles); Figure S2: Shift factors as a function of cupin-1.2 (a) and vicilin (b) concentration; Figure S3: Kinetic dependences of the real part of the surface elasticity of recombinant cupin-1.2 (a) and vicilin (b) solutions in 8M urea at the concentrations of 1 (olive triangles), 2.5 (magenta triangles), 5 (cyan diamonds), 10 (blue triangles), 20 (green triangles), 50 (red circles), and 100 mg/L (black squares); Figure S4: The ratio between imaginary and real parts of surface elasticity of cupin-1.2 (a) and vicilin (b) solutions in 8M urea at the concentrations of 1 (olive triangles), 2.5 (magenta triangles), 5 (cyan diamonds), 10 (blue triangles), 20 (green triangles), 50 (red circles), and 100 mg/L (black squares) as a function of the surface pressure; Figure S5: AFM images of cupin-1.2 (a–c) and vicilin (d–f) spread layers on water surface at different compression steps: a and d—before the compression; b and e—layers in the region of the elasticity maximum; c and f—layers in the region of the elasticity minimum. The layer average thickness is 1.4 (a), 0.79 (b), 1.1 nm (c) for cupin-1.2 layers, and 0.69 (d), 1.3 (e) and 1.1 nm (f) for vicilin layers. The layer average roughness is 0.3 (a), 0.2 (b) and 0.4 nm (c) for cupin-1.2 layers, and 0.3 (d), 0.5 (e) and 0.3 nm (f) for vicilin layers; Figure S6: Kinetic dependences of the surface pressure of cupin-1.2 (a) and vicilin (b) solutions in 8M urea at concentrations of 1 (olive triangles), 2.5 (magenta triangles), 5 (cyan diamonds), 10 (blue triangles), 20 (green triangles), 50 (red circles), and 100 mg/L (black squares). Time is presented in square root scale.

## Abbreviations

The following abbreviations are used in this manuscript:

AFM	Atomic force microscopy
GRAVY	Grand average of hydropathy
AI	Aliphatic index
