# Supplementary material for: Surface Properties of Recombinant Pea Vicilin and Cupin-1.2 Solutions in 8M Urea
Source: Polymers (Basel). 2025 Sep 11;17(18):2463. doi: 10.3390/polym17182463 (PMC12473962; doi:10.3390/polym17182463)
Supplement: Supplementary file 1 [file polymers-17-02463-s001.zip › polymers-3810575-supplementary.pdf]

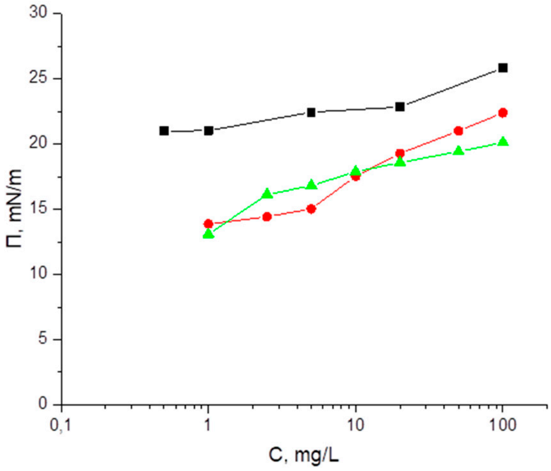

Figure S1. Surface pressure at surface age of 420 min as a function of the concentration of cupin-1.1 (black squares), cupin-1.2 (red circles), and vicilin (green triangles). The data for cupin-1.1 solutions are replotted from [30].

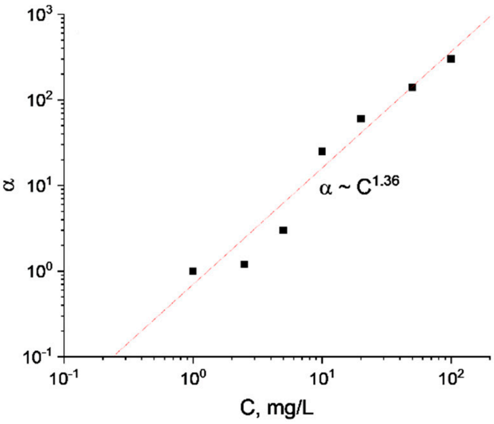

(**a**)

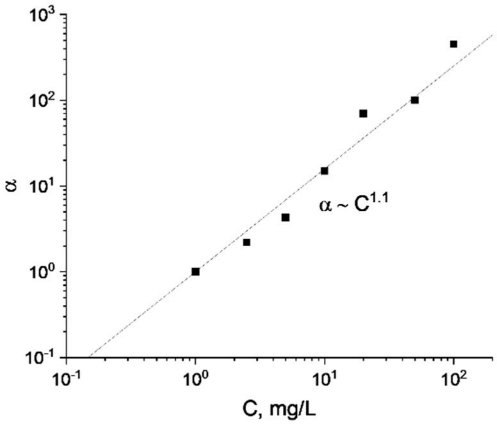

(**b**)

Figure S2. Shift factors as a function of cupin-1.2 (a) and vicilin (b) concentration.

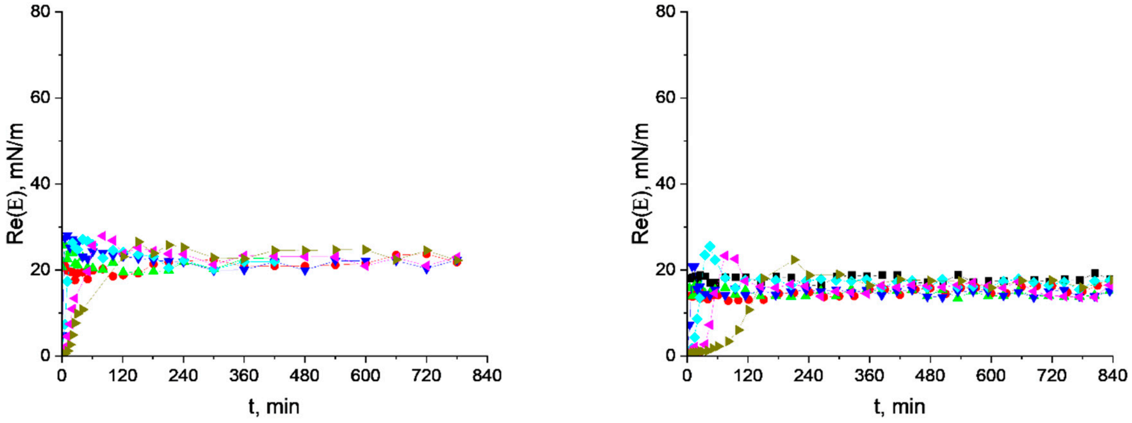

(**a**) (**b**)

Figure S3. Kinetic dependences of the real part of the surface elasticity of recombinant cupin-1.2 (a) and vicilin (b) solutions in 8M urea at the concentrations of 1 (olive triangles), 2.5 (magenta triangles), 5 (cyan diamonds), 10 (blue triangles), 20 (green triangles), 50 (red circles), and 100 mg/L (black squares).

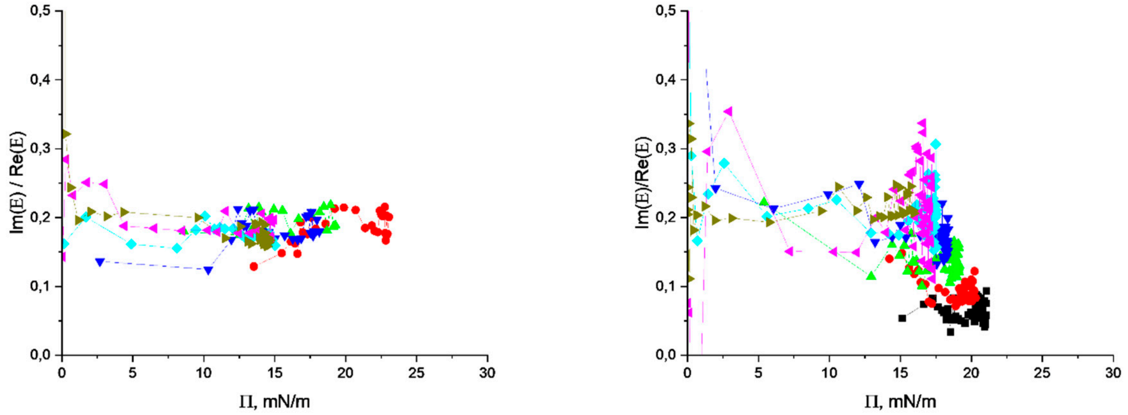

(**a**) (**b**)

Figure S4. The ratio between imaginary and real parts of surface elasticity of cupin-1.2 (a) and vicilin (b) solutions in 8M urea at the concentrations of 1 (olive triangles), 2.5 (magenta triangles), 5 (cyan diamonds), 10 (blue triangles), 20 (green triangles), 50 (red circles), and 100 mg/L (black squares) as a function of the surface pressure.

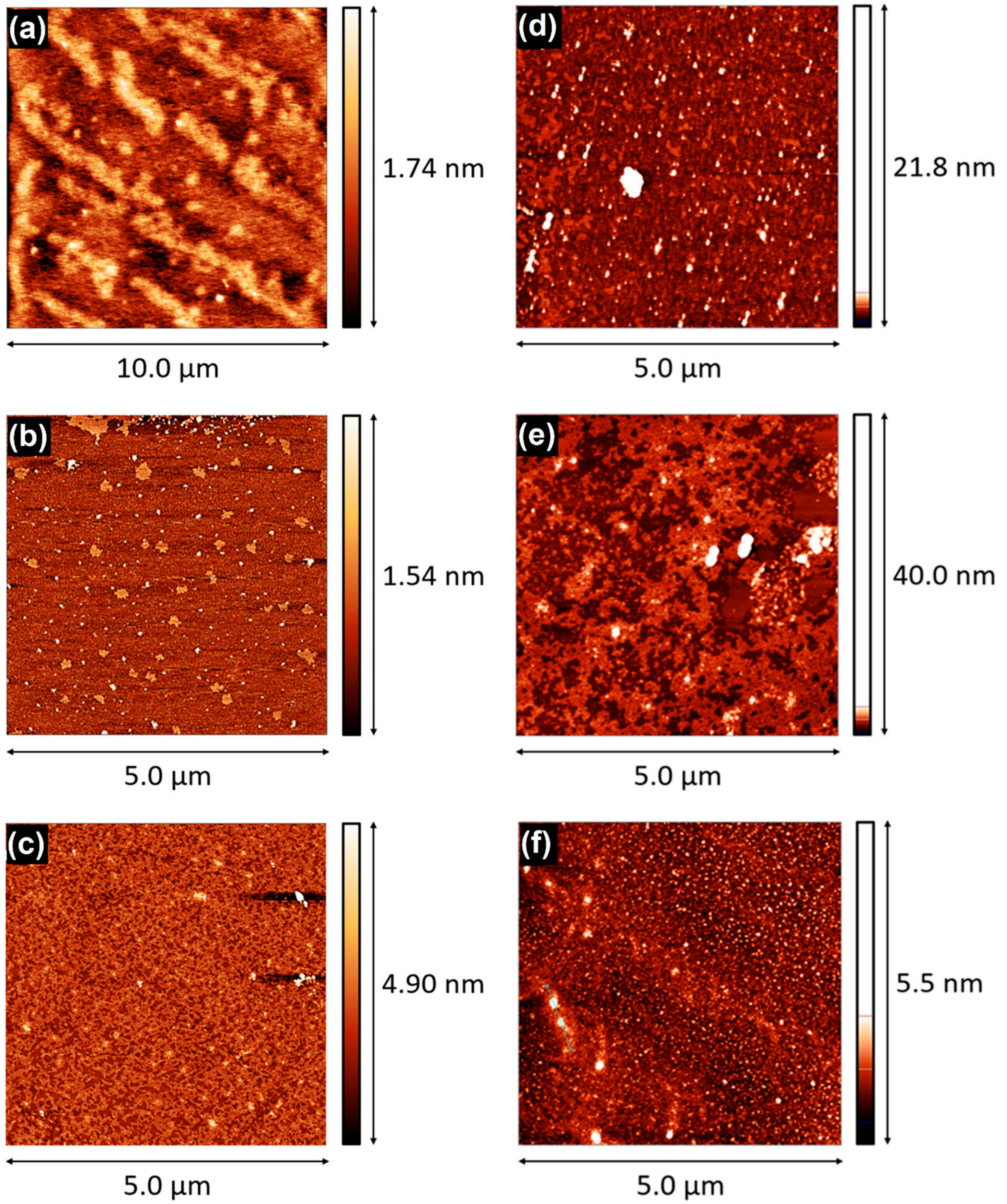

Figure S5. AFM images of cupin-1.2 (a-c) and vicilin (d-f) spread layers on water surface at different compression steps: a and d – before the compression; b and e – layers in the region of the elasticity maximum; c and f – layers in the region of the elasticity minimum. The layer average thickness is 1.4 (a), 0.79 (b), 1.1 nm (c) for cupin-1.2 layers, and 0.69 (d), 1.3 (e) and 1.1 nm (f) for vicilin layers. The layer average roughness is 0.3 (a), 0.2 (b) and 0.4 nm (c) for cupin-1.2 layers, and 0.3 (d), 0.5 (e) and 0.3 nm (f) for vicilin layers.

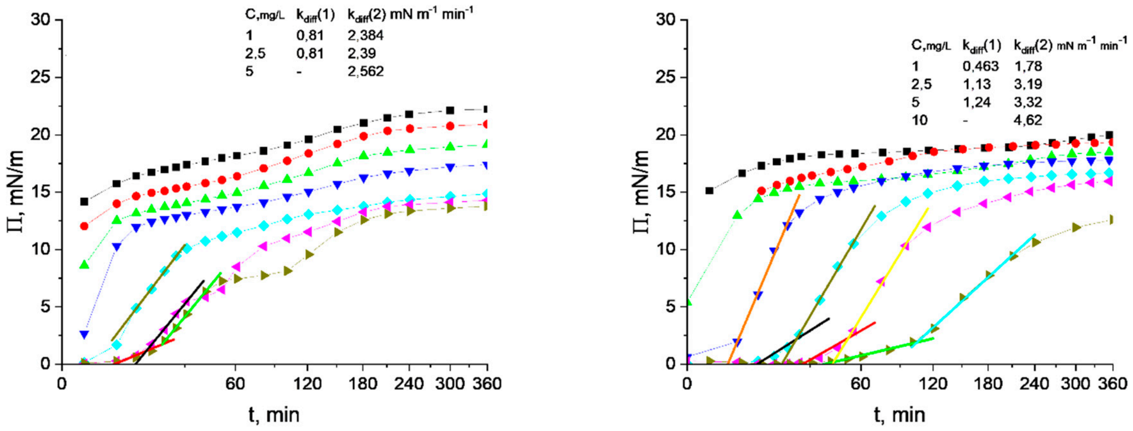

(**a**) (**b**)

Figure S6. Kinetic dependences of the surface pressure of cupin-1.2 (a) and vicilin (b) solutions in 8M urea at concentrations of 1 (olive triangles), 2.5 (magenta triangles), 5 (cyan diamonds), 10 (blue triangles), 20 (green triangles), 50 (red circles), and 100 mg/L (black squares). Time is presented in square root scale.
